# Rational Method for Structural Simplification as Key Step in Hit Discovery: The Case of FGFR2 and IGF1R Dual Inhibitors

**DOI:** 10.3390/ijms26094457

**Published:** 2025-05-07

**Authors:** Endika Torres-Urtizberea, José I. Borrell, Raimon Puig de la Bellacasa, Roger Estrada-Tejedor

**Affiliations:** Grup de Química Farmacèutica, IQS School of Engineering, Universitat Ramon Llull, Via Augusta 390, E-08017 Barcelona, Spain; endika.torres@iqs.url.edu (E.T.-U.); jose.borrell@iqs.url.edu (J.I.B.); raimon.puig@iqs.url.edu (R.P.d.l.B.)

**Keywords:** QSAR, structure complexity, hit discovery

## Abstract

In the classic medicinal chemistry hit discovery procedure, large virtual libraries undergo different filtering and prediction steps until a small group of molecules is selected for their subsequent synthesis and biological testing. The starting molecular libraries can easily be composed of millions of molecules, hindering the selection of the most representative and promising compounds. Moreover, the resulting molecular systems tend to be overcomplex structures, hardly attainable, and often involve extrapolations of the prediction models used. We present a rational-based method to reduce the structural complexity of molecular candidates without compromising their biological activity, improving the attainability and efficiency of hit discovery. This approach has been successfully applied to identify potential tyrosine kinase dual inhibitors against Fibroblast Growth Factor Receptor 2 (FGFR2) and Insulin-Like Growth Factor 1 Receptor (IGF1R), a set of overexpressed proteins in different cancers, such as pancreatic ductal adenocarcinoma (PDAC).

## 1. Introduction

**Figure 1:**
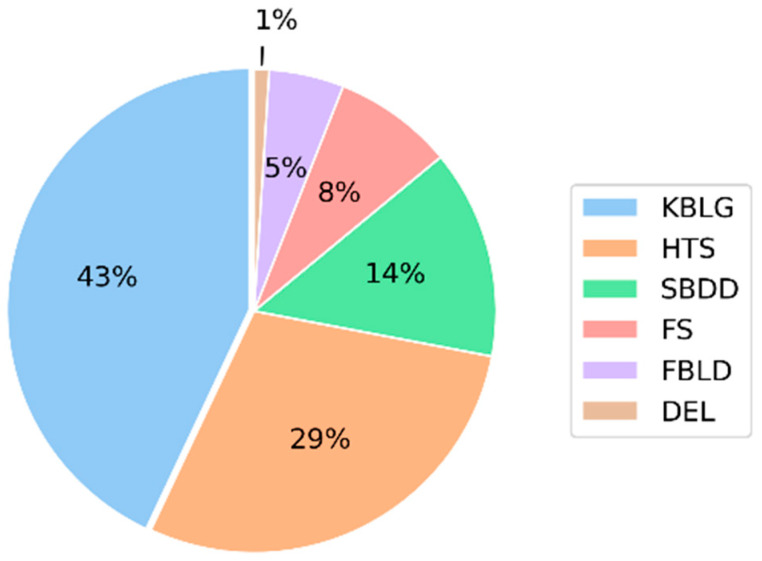
Percentage of lead generation sources published in J. Med. Chem between 2016 and 2017 [5]. Knowledge-based lead generation (KBLG), high-throughput screening (HTS), structure-based drug design (SBDD), focused screening (FS), fragment-based lead generation (FBLG), DNA encoded libraries (DEL).

In the drug development process, choosing the strategy for lead discovery is often a crucial step, as it will determine the starting point of the whole process. Such strategies are often based on high-throughput screening (HTS) [1], structure-based drug design (SBDD) [2], fragment-based lead generation (FBLG) [3] or the most used, knowledge-based lead generation (KBLG) [4]. While the selection of a strategy is driven by several factors, KBLG has been the most used method overall, as it allows one to skip the hit identification step, increasing the process’ efficacy while reducing the costs (Figure 1).

Although KBLG has been successful in identifying new leads [5], it requires a large number of very detailed, high-quality data, which, in many cases, are not available in open databases. For this reason, in-house databases are commonly used for hit selection, allowing the generation of tailored structures that may align more closely with the research strategy of a given research group. At the same time, this ensures the data’s sensitivity and that the intellectual property remains within the group, while increasing the novelty of future discoveries. The generation of such hit selection often begins by synthesizing and testing a set of molecules [6], These, ideally, would be easily synthesizable while having a high probability of showing the desired activity, generating as many hits as possible in the minimum time possible. For this, the selection of the structures to be synthesized should consider both structural diversity and synthetic feasibility, as they will set the foundations for lead generation.

**Figure 2:**
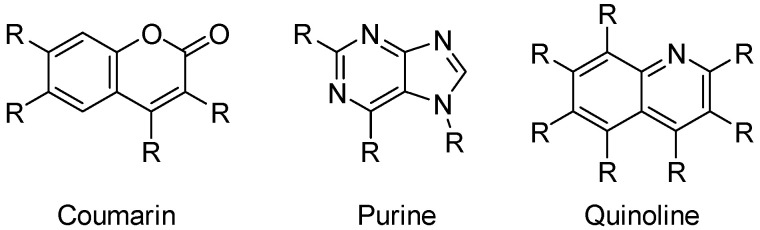
Examples of some privileged scaffolds and their positions amenable to functionalization.

A popular means of generating hit candidates is ligand-based drug design (LBDD) approaches [7], where predictive models allow the determination of the best candidates to be synthesized from a previously generated pool of structures. This pool is generated by centering the structure generation around a privileged scaffold [8], which, thanks to its various substitution points, eases the creation of numerous and diverse structures by applying combinatorial approaches in which an independent number of diverse substituents is proposed for each position amenable to functionalization (Figure 2). However, these approaches are often affected by the generation of highly complex structures, which hinders the progression of the hit discovery process [9], as they would require longer synthetic paths. This situation leads to the selection of less complex structures, often consisting of non-relevant molecules, neglecting relevant information from complex structures.

In this work, we present a rational methodology to reduce the structural complexity of drug candidates, removing unnecessary structural features that may pose synthetic challenges while keeping relevant structural information by considering their effects on the predicted activity. This would expedite greatly the hit discovery process in small research groups, not only reaching the hit-to-lead stage sooner but also allowing the use of in-house KBLG once the hit library grows sufficiently. Moreover, the suggested methodology lacks human bias, allowing the selection of structures that may not have been explored otherwise, increasing the chemical space coverage while maintaining high predicted activity.

**Figure 3:**
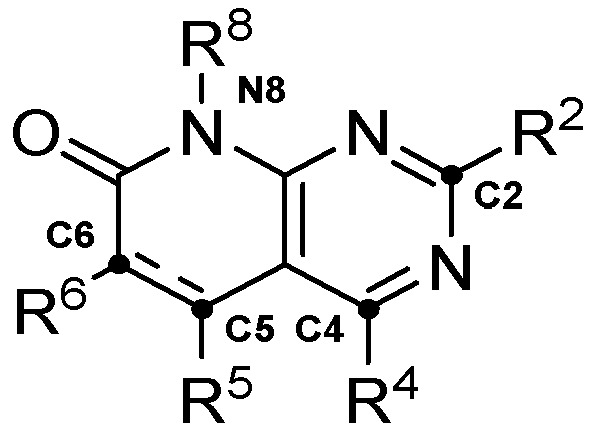
Main substitution points in the pyrido[2,3-*d*]pyrimidine scaffold.

The use and validation of the method is demonstrated within the context of a project focused on developing dual tyrosine kinase inhibitors (TKI) targeting Fibroblast Growth Factor Receptor 2 (FGFR2) and Insulin-Like Growth Factor 1 Receptor (IGF1R), two proteins that have previously been identified as promising targets against PDAC [10,11]. The simultaneous inhibition of these proteins has been shown to reduce both tumor progression and drug resistance development [12,13,14]. At the same time, the pyrido[2,3-*d*]pyrimidine scaffold was used as the core for the generation of the combinatorial library, given its high degree of tunability and biologically active examples [15,16,17,18,19,20,21] (Figure 3).

## 2. Results and Discussion

### 2.1. QSAR Model Performance

Following the methodology stated at Materials and Methods, the created prediction models performed outstandingly, achieving Q^2^ values of above 0.7 and requiring less than 70 features (Table 1).

#### Application of the Prediction Model on the Test Set

**Figure 4:**
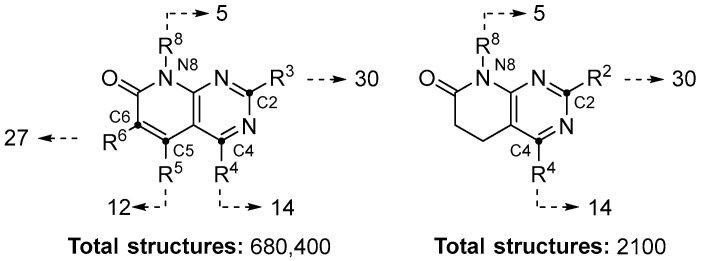
Number of substituents per pyrido[2,3-*d*]pyrimidine scaffold position.

The generated model was applied on a test set composed of the combinatorial library obtained by substituting the pyrido[2,3-*d*]pyrimidine scaffold’s diversifiable points (Figure 4). The final combinatorial library included a total number of 682,500 different molecules.

Due to the structural diversity of the compounds included in the combinatorial library, some of them fell outside the applicability range of the prediction models, as their feature values did not fit within the margins set by the training dataset.

**Figure 5:**
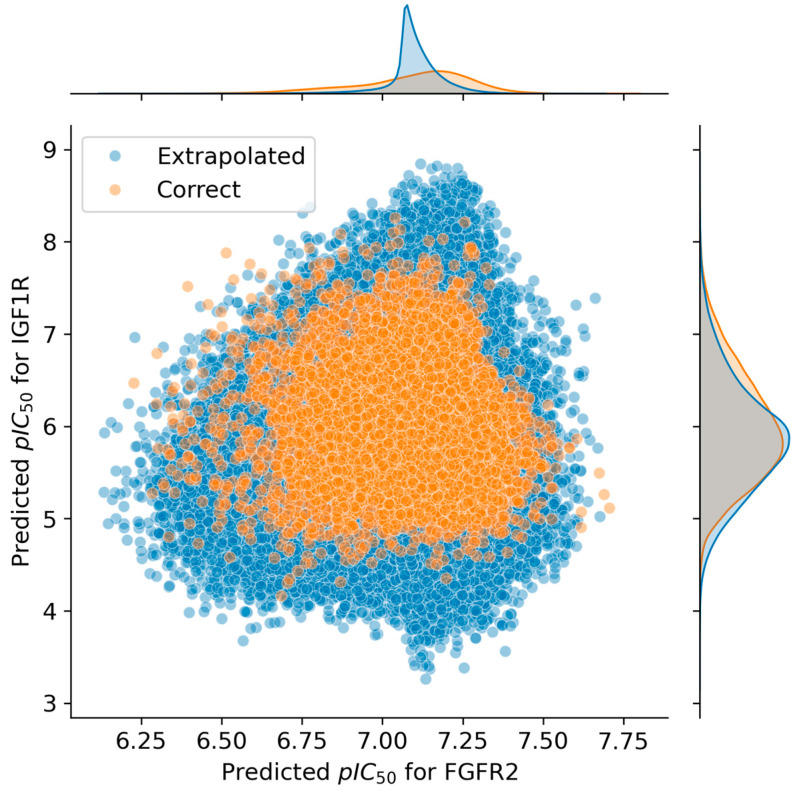
Distribution analysis of the predicted pIC_50_ values against FGFR2 and IGF1R. Molecules with features included in the range of the training set are shown in orange, while compounds that are supposedly extrapolating are shown in blue.

The database was consequently divided into two groups: those whose prediction was considered extrapolated and those whose predictions were not. Therefore, the predicted activity distribution of each group was plotted (Figure 5), which showed that extrapolated predictions were found throughout the whole observed activity value range, indicating a lack of extrapolation bias. Considering this, using the whole unfiltered database would have given as much information as possible without compromising the quality of the simplification.

**Figure 6:**
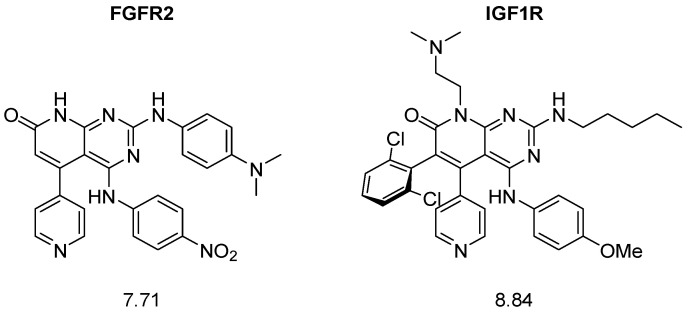
Example structures with high predicted pIC_50_ values for each protein.

A closer examination of the most active molecules confirmed the first part of the proposed hypothesis: due to the nature of the combinatorial structure generation, the best predicted structures showed great complexity due to their high degree of substitution (Figure 6).

The synthesis of these structures would require, in the best case, more than 8 steps, becoming a very costly means of generating hit molecules.

**Figure 7:**
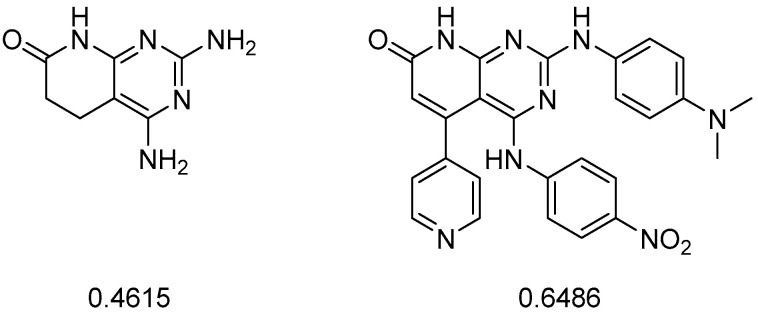
The synthetic feasibility molecular descriptor (rsynth), calculated in MOE, leads to a value between 0 and 1 (where 0 corresponds to a not synthetically feasible structure and 1 means that it is fully feasible). For instance, this descriptor considered less feasible a pyrido[2,3-*d*]pyrimidine structure that was obtained in a single step compared to another that required more than eight steps.

Following our hypothesis, if we were able to rank the best structures by both synthetic feasibility and predicted activity, much more efficient candidates could be selected. The problem with this type of metric is its lack of consistency, being very dependent on the type of structure used (Figure 7).

It is challenging to define synthetic feasibility as it should consider the reaction steps, reaction complexity and reagent availability, among others [22]. While these aspects are strongly dependent on the used scaffold and substituents, generally, one can understand that the more decorated a scaffold is, the longer the synthetic path will likely be. Based on this, we defined synthetic feasibility solely as a function of the number of substitutions in the scaffold, meaning that reducing the number of substitutions would increase the synthetic feasibility.

In order to conduct the simplification of the structural complexity in a rational way, we proposed taking advantage of SAR analysis tools, as they would allow us to eliminate those substituents with weaker relations to the predicted activity.

### 2.2. SAR-Based Structural Simplification

SAR methodologies are the foundation of drug design [23], allowing a systematic approach to understanding how the chemical structures of molecules influence their biological activity.

Although this type of methodology is not usually used with predicted activity values, it could help to assess the influence of the substituents on the prediction model. SAR analysis could be therefore used to identify the most frequent substituents among the compounds with higher predicted activity. Thus, one could reduce the structural complexity by focusing on structurally simple structures bearing the privileged substituents identified from compounds with the highest predicted activity.

This procedure would yield structures with reduced predicted activity, but it should also allow the fast exploration of several substituents that could point towards more complex structures requiring longer synthetic paths.

At the same time, while this method did not consider the synergistic effect between multiple substitutions, since the substituents were chosen based on the frequency of appearance, we expected that these substituents could maintain their activity, even when different substituents were present in the scaffold, making them privileged. Structures derived from these could be considered good hit candidates as they would still maintain the possibility of further modification in the following hit-to-lead steps.

#### Finding Privileged Substituents

Using MOE’s SAR analysis tool (SAReport), the 4000 structures with the best average predicted activity against both FGFR2 and IGF1R were analyzed, defining pIC50 > 7 as the threshold to identify the most active molecules.

**Figure 8:**
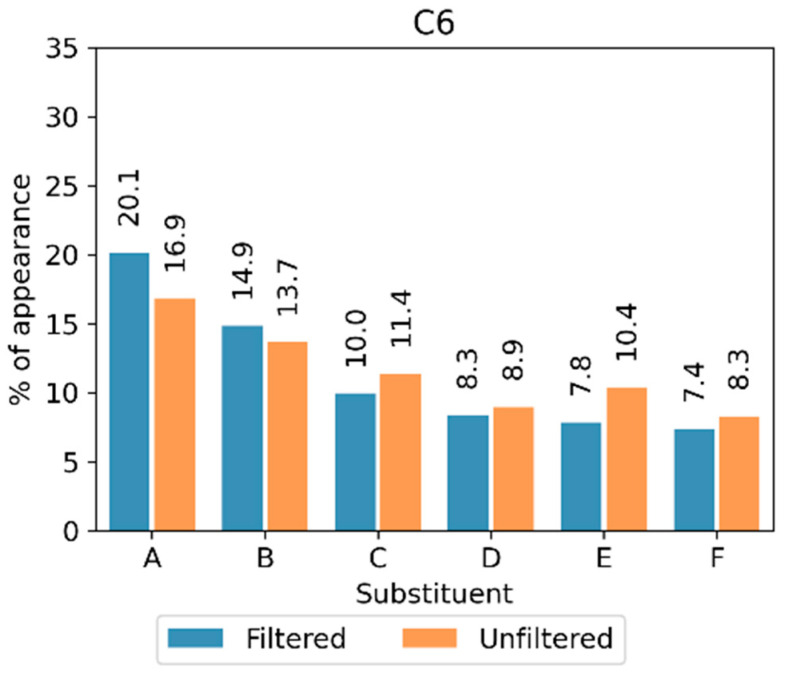
Percentage of appearance of the most popular substituents at the C6 position when removing the compounds that permit the extrapolation of the prediction model (filtered) or analyzing the entire chemical library (unfiltered). The different structural fragments are labeled with capital letters (A–F).

The results showed that no significant differences existed between the privileged substituents found among both the extrapolating and the non-extrapolating compounds. This further supported the idea that extrapolation was not an issue in this case (Figure 8).

**Figure 9:**
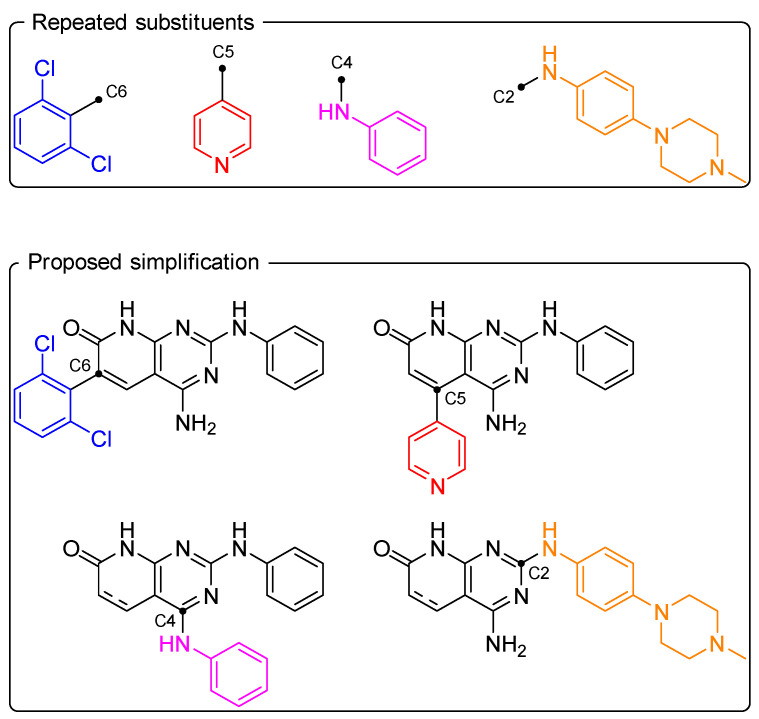
Visual representation of the simplified structure generation.

Using the privileged substituents for each position, one or more simplified pyrido[2,3-*d*]pyrimidines were generated, where only the given substituent was maintained in the structure (Figure 9).

In this way, 94 simplified structures were generated. While analyzing the number of molecules and substituents throughout the whole process, the initial molecule number was reduced by 99.98%, and, among the different substituents generating the combinatorial database, only 25% were considered privileged (Table 2).

**Figure 10:**
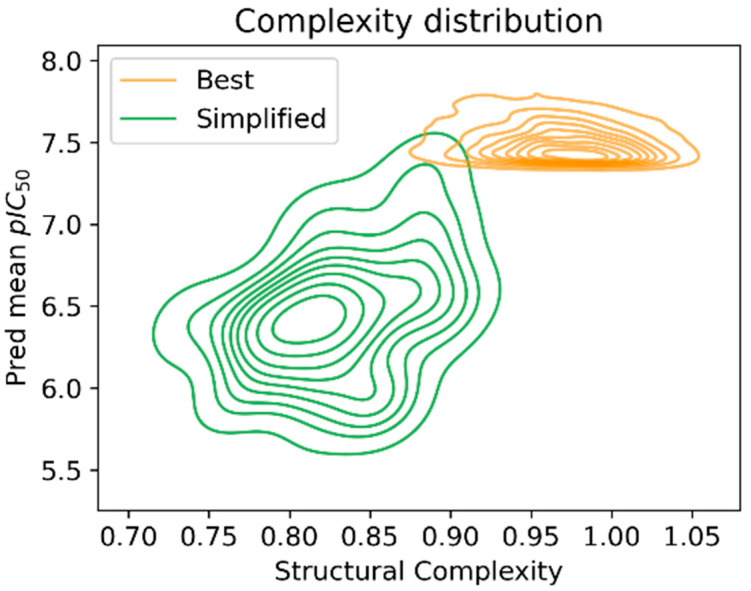
Simplification distribution of the simplified structures and the 4000 best predicted ones.

To quantify the complexity reduction achieved by the suggested strategy, the complexity parameter defined by DataWarrior [24] was calculated. These values are represented in the kernel density estimation plot (Figure 10), where the reduced structural complexity of the simplified set of molecules is apparent. At the same time, as was expected, this complexity reduction came with a cost in the predicted pIC_50_; still, their reduced synthetic paths should compensate for this, as, once their real activity is measured, it would allow us to identify which complex structures are worth investing resources in.

**Figure 11:**
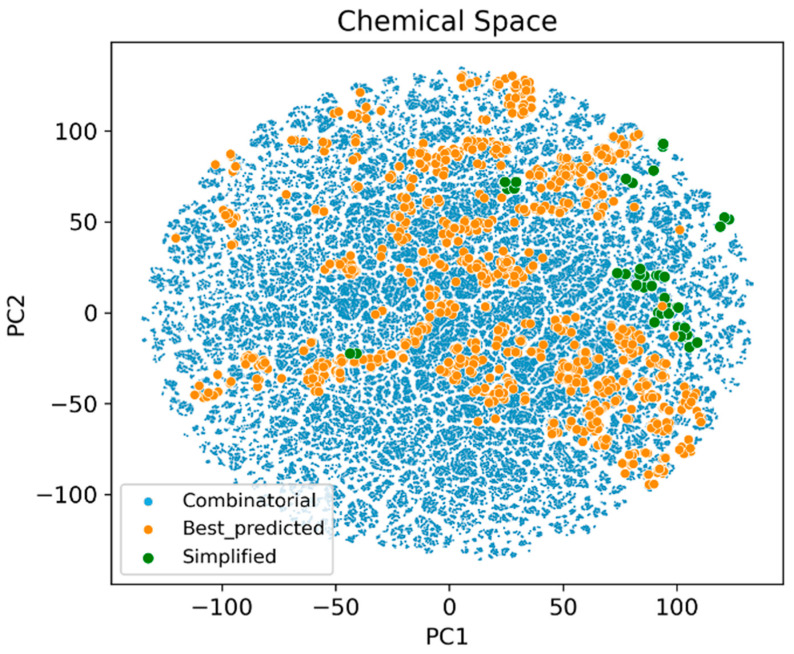
TSNE plot of the different molecule sets used in the structural simplification process.

To further analyze these results, the t-distributed stochastic neighbor embedding (TSNE) algorithm was used to plot the chemical spaces of the three molecule sets: the initial combinatorial database, the 4000 best compounds used in the SAReport and the simplified 94 molecules (Figure 11).

It can be seen how most of the 94 simplified structures are located in areas covered by the best 4000 compounds, indicating that they still maintained a favorable activity profile. At the same time, their peripheral location relative to the orange molecules shows also their structural differences.

### 2.3. Final Candidate Selection

The 94 simplified structures were ranked based on Pareto optimization metrics. This method is widely used for the optimization of multi-objective virtual screening [25], where molecules that form or are close to the Pareto front receive higher scores, balancing the predicted value against each target.

Based on this, the highest-scoring 17 compounds were selected, taking into account the privileged substituent diversity, thus selecting as many different substitutions as possible (Table 3).

From this compound selection, it could be seen that the SAR-based structural simplification led us towards structures that would not have been selected otherwise, due to them having too low activity or being hidden behind a huge database, as none of them were among the best 1000 compounds.

For the external validation of the hypothesis, the two best-predicted non-simplified compounds were selected (Table 4).

### 2.4. Synthesis of the Candidates

The chosen candidates were synthesized following different synthetic paths, depending on the substituted position of the pyrido[2,3-*d*]pyrimidines, based on methodologies developed in the group. To illustrate this, the synthesis of three key examples, IQS224, IQS226 and IQS229, is disclosed.

#### 2.4.1. Synthesis of IQS226

**Figure 12:**
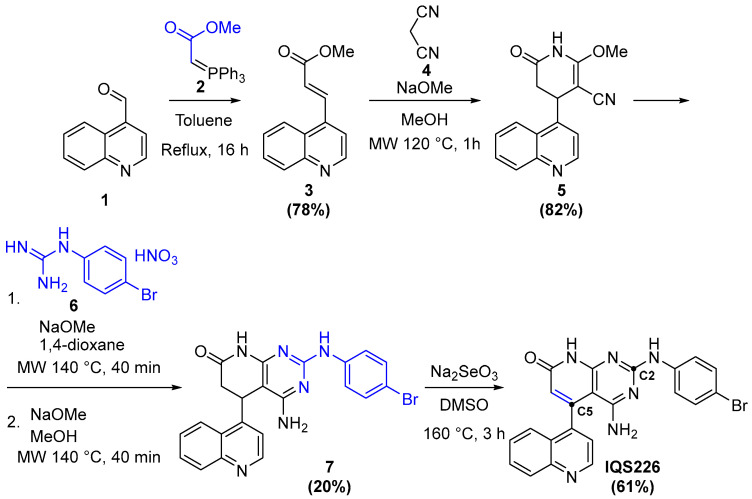
The followed synthetic path towards IQS226.

Generally, C2- and C5-substituted compounds begin with the formation of a substituent bearing acrylate **3** by a Wittig reaction; it is then heated with malononitrile (**4**) and NaOMe assisted by microwave irradiation. The formed pyridone **5** is then reacted with a 4-bromophenylguanidine nitrate (**6**), which, over two steps [19], yields the pyrido[2,3-*d*]pyrimidine scaffold **7**. Finally, IQS226 is obtained after the dehydrogenation of **7** and its reaction with Na_2_SeO_3_ at high temperatures [26] (Figure 12).

#### 2.4.2. Synthesis of IQS229

**Figure 13:**
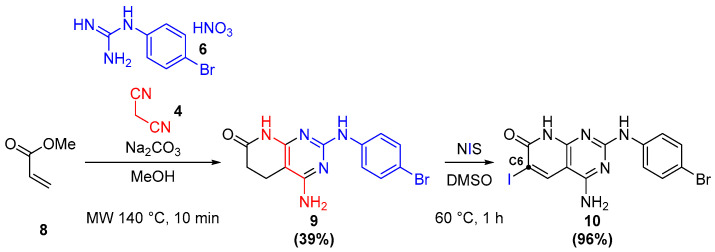
Formation of the common intermediate **10**.

In the case of C2 and C6 substitution, the synthesis of the common intermediate **10** allowed a much more efficient route towards the candidates. Molecule **10** is obtained by the path shown in Figure 13, where, after the microwave-assisted one-pot formation of the pyrido[2,3-d]scaffold **9** [27], its reaction with NIS leads to dehydrogenation and iodination, introducing a key iodine in the C6 position.

**Figure 14:**
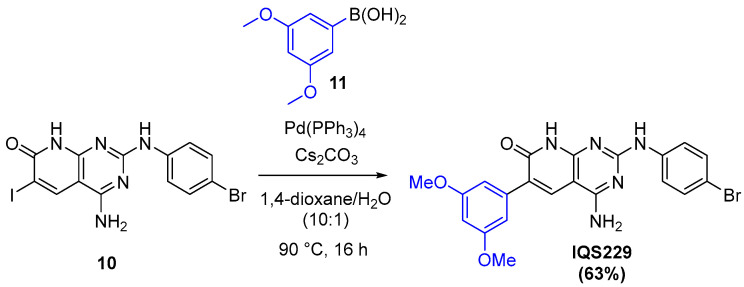
Suzuki cross-coupling reaction for the formation of IQS229.

From this point, different organometallic reactions allow the late-stage introduction of diversity into the structure [27]. Specifically, the selective palladium-catalyzed Suzuki cross-coupling depicted in Figure 14 gave IQS229 in a single step.

#### 2.4.3. Synthesis of IQS224

**Figure 15:**
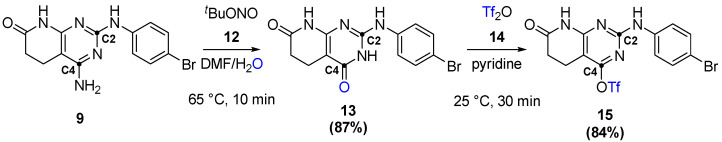
Introduction of a leaving group at the C4 position of the pyrido[2,3-*d*]pyrimidine.

C2- and C4-substituted structures follow a similar strategy as the one above, where a leaving group is introduced in the C4 position by the reactions shown in Figure 15. Here, the previously obtained **9** would be reacted using Sandmeyer-like conditions, forming compound **13**. This would be reacted with triflic anhydride (**14**), allowing the introduction of the pseudohalogen at the C4 position.

**Figure 16:**
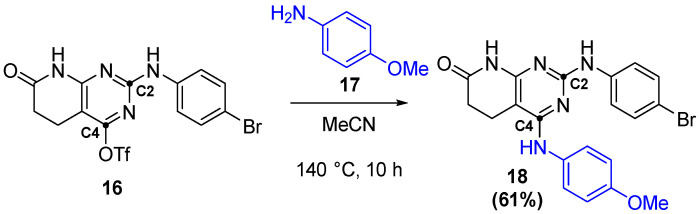
Formation of IQS224 by a S_N_Ar reaction.

This leaving group would then be substituted by either organometallic reactions [20] or, in the case of IQS224, by a direct S_N_Ar (Figure 16).

### 2.5. Biological Evaluation

**Figure 17:**
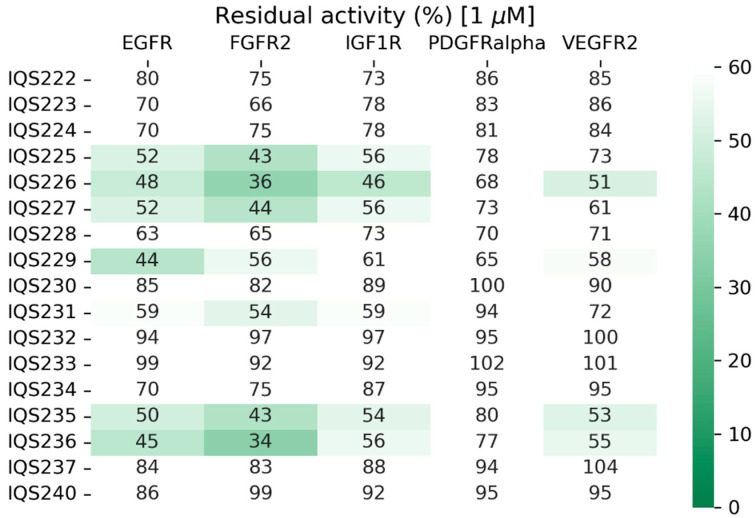
Residual activity percentages of the target proteins when treated with 1 μM of the simplified inhibitors in an enzymatic assay.

The validation of the process was performed by measuring each molecule’s TKI activity through an enzymatic assay against our targeted proteins (Figure 17).

From the 17 synthesized molecules, seven of them inhibited both FGFR2 and IGF1R with an IC_50_ value close to 1 μM (residual activity below or very close to 50%) and thus were considered acceptable hits. This meant that the success rate of the methodology described was close to 50% in both proteins, making it a very effective method of hit selection.

In addition, in the case of IGF1R, the predicted and measured activity values achieved high Spearman correlation values (ρ = 0.64), further supporting the applicability of the methodology.

**Figure 18:**
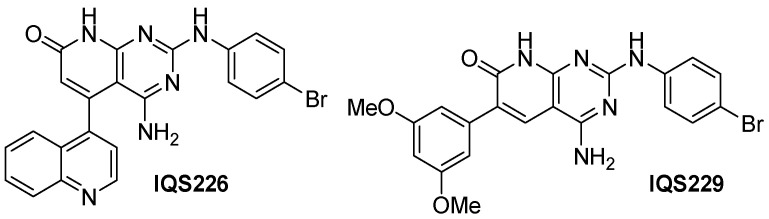
Example structures of the simplified active candidates.

Both IQS226 and IQS229, disclosed previously, are among the seven active candidates (Figure 18).

**Figure 19:**
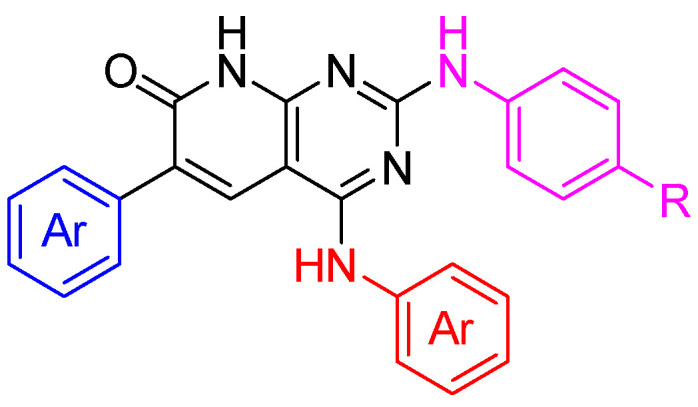
General structures of the non-simplified candidates. Each structure has a molecular weight above 570 Da.

To further validate the hypothesis of this work, two non-simplified candidates (Figure 19) were synthesized following non-published methodologies.

**Figure 20:**
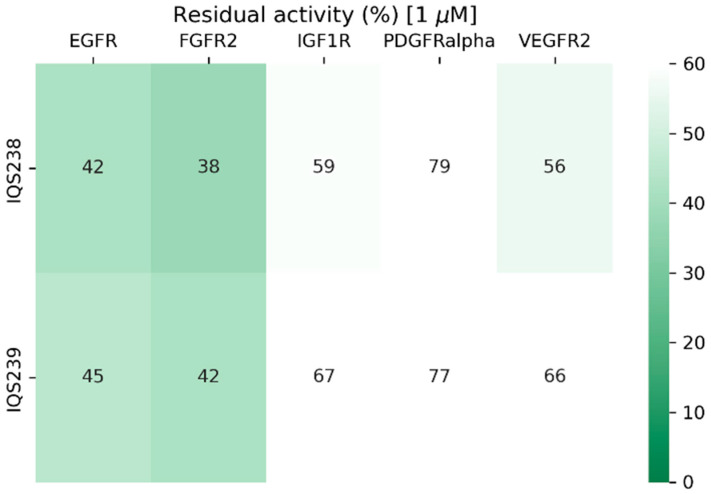
Residual activity percentages of the target proteins when treated with 1 μM of complex inhibitor (IQS238 and IQS239) in an enzymatic assay.

These candidates (IQS238 and IQS239) showed very similar activity (Figure 20) while requiring up to eight synthetic steps to obtain and having more limited structural diversity.

Considering these results, in an early hit discovery scenario, synthesizing a diverse group of molecules is preferred even if the success rate is lower, as both active and inactive molecules will serve as information for more active structures.

## 3. Computational Methodology

### 3.1. Datasets

QSAR models were trained using the previously curated ChEMBL database [28] (n_FGFR2_ = 687, n_IGF1R_ = 816), with known pIC_50_ values (measured in single protein format assays), where duplicate information and non-exact or missing activity values were removed.

Smilib v2.0 [29] was used to create the combinatorial library used as a test set. Independent SMILES libraries for the different substituents were created considering the synthetic feasibility of each independent substitution, the structural diversity of the groups and the availability of reagents (the list of the different substituents is available in the Supplementary Materials).

### 3.2. Feature Selection

All structural-related calculations were carried out using the Molecular Operating Environment (MOE) [30]. All 2D molecular descriptors (n = 206) were calculated and used as starting features.

The in-house feature selection method consisted of merging three well-described feature selection methods, compensating for each one’s limitations while yielding an efficient and effective new hybrid feature selection methodology.

**Figure 21:**
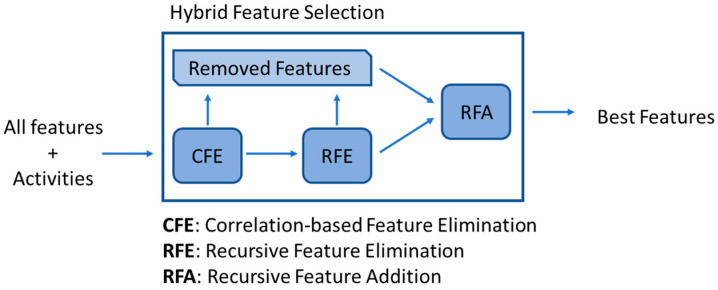
In-house feature selection methodology, where CFE, RFE and RFA are combined.

The method consisted of correlation-based feature elimination (CFE), which reduced the initial set of features based on their correlations with the pIC_50_ value, removing those with a correlation below 0.3. The remaining features were further reduced by recursive feature elimination (RFE), where all those features whose elimination would increase the model’s performance were removed. Finally, all removed features were retested using recursive feature addition (RFA), where each was added to the remaining set of features, maintaining those that improved the model’s performance (Figure 21).

This method was able to outperform the three algorithms that it was based on in terms of feature reduction, the obtained precision and the calculation times.

### 3.3. QSAR Model Creation Methodology

The quantitative structure–activity relationship (QSAR) prediction models were generated using Python’s Scikit-Learn library [31], where the support vector machines (SVM) algorithm was chosen based on previous group experience.

Its hyperparameters were selected using grid search optimization, using the selected features (Supplementary Materials).

The models’ precision was measured using leave one out cross-validation (LOO-CV) and validated using 5-fold and 10-fold cross-validation (CV5 and CV10) and y-randomization (Supplementary Materials).

### 3.4. Structural Simplification Method

**Figure 22:**
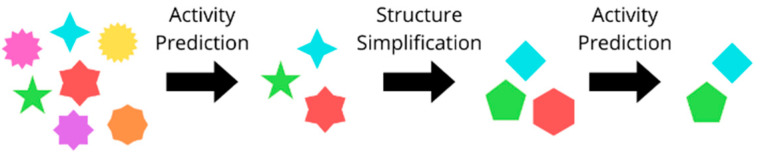
Visual representation of the structure simplification procedure. Each polyhedron depicts a molecule, where a reduction in vertex exemplifies the simplification process.

The molecules with the best predicted pIC_50_ values were simplified following a multistep method, where an automatized structure–activity relationship (SAR) analysis tool (SAReport, available in MOE 2022 [30]) allowed us to highlight the structural decorations responsible for the predicted activity, which served as the basis for the generation of a new group of structures bearing fewer substituents. Among these, those with the best predicted activity were selected, yielding a selection of simplified molecules (Figure 22).

## 4. Conclusions

Starting with a combinatorial library containing close to 700,000 structures, we developed a methodology that allowed the rational selection of 17 simplified molecules. Compared to non-simplified candidates, these were obtained using divergent and efficient synthetic methodologies, yielding a moderate number of molecules in a fast manner, while maintaining a high rate of success. The proposed methodology was validated using two well-established kinases, FGFR2 and IGF1R, whose roles have been linked in various pathological conditions (e.g., pancreatic ductal adenocarcinoma, PDAC). This allowed us to discover seven novel active inhibitors, while uncovering various privileged functional groups, such as the disclosed 2,4-methoxyphenyl group in IQS229, key in the discovery of Futibatinib, a commercial FGFR inhibitor [32]. These results not only show the efficacy of this structural simplification methodology but also demonstrate its potential to be a standard practice in hit discovery programs, as it allows the fast identification and development of bioactive compounds. At the same time, the fact that it was used to develop multitarget inhibitors shows the process’ flexibility, as it could be applied to diverse drug design campaigns.

## Figures and Tables

**Table 1:** Correlation coefficient values for the validation of the SVM model against the target proteins, in the training set (R^2^ self) and the validation set: LOO (Q^2^), CV5 (R^2^ CV5), CV10 (R^2^ CV10) and y-randomization (y-rand).

Target	R^2^ (Self)	Q^2^	R^2^ (CV5)	R^2^ (CV10)	y-Rand	Features
FGFR2	0.946	0.733	0.698	0.720	−0.285	69
IGF1R	0.925	0.739	0.695	0.710	−0.352	51

**Table 2:** Summary of the number of substituents and molecules in the simplification process.

	Starting Database	SAReport	Simplification
C2 substituents	30	20	5
C4 substituents	14	9	4
C5 substituents	12	10	5
C6 substituents	27	25	7
N8 substituents	5	5	4
TOTAL	680,400	4000	94

**Table 3:** Summary of the selected candidates after the structure simplification process. The last two columns show their ranks among the final 94 simplified compounds and the original combinatorial database based on their mean predicted pIC_50_.

Name	IGF1R	FGFR2	Mean	Pareto Value	Rank Combinatorial
IQS227	7.81	7.08	7.44	92	1978
IQS228	7.55	6.96	7.25	71	5479
IQS229	7.53	6.95	7.24	68	5943
IQS236	7.11	6.95	7.03	63	22,997
IQS222	6.12	7.11	6.61	59	214,246
IQS226	6.46	6.84	6.65	48	110,333
IQS231	6.82	6.76	6.79	40	49,587
IQS232	6.02	6.86	6.44	39	258,649
IQS230	6.40	6.77	6.58	37	125,595
IQS240	6.01	6.83	6.42	36	263,397
IQS237	6.23	6.78	6.51	34	174,159
IQS224	7.48	6.66	7.07	30	6987
IQS235	6.94	6.66	6.80	27	36,502
IQS233	6.25	6.69	6.47	26	168,728
IQS223	6.49	6.58	6.49	21	104,524
IQS234	6.38	6.49	6.43	13	128,756

**Table 4:** Predicted pIC_50_ values of the two best non-simplified compounds.

Name	IGF1R	FGFR2	Mean	Rank Combinatorial
IQS238	8.79	7.23	8.01	1
IQS239	8.82	7.17	8.00	2

## Data Availability

The data presented in this study are available on request from the corresponding author due to privacy.

## Supplementary Materials

The following supporting information can be downloaded at: https://www.mdpi.com/article/10.3390/ijms26094457/s1 [33,34].

## Abbreviations

The following abbreviations are used in this manuscript:

CFE	Correlation-Based Feature Elimination
CV	Cross-Validation
DEL	DNA-Encoded Libraries
DMF	Dimethylformamide
FBLG	Fragment-Based Lead Generation
FGFR2	Fibroblast Growth Factor Receptor 2
FS	Focused Screening
HTS	High-Throughput Screening
IGF1R	Insulin-Like Growth Factor 1 Receptor
KBLG	Knowledge-Based Lead Generation
LBDD	Ligand-Based Drug Design
LOO	Leave One Out
NIS	*N*-Iodosuccinimide
MOE	Molecular Operating Environment
MW	Microwave
PDAC	Pancreatic Ductal Adenocarcinoma
QSAR	Quantitative Structure–Activity Relationship
RFA	Recursive Feature Addition
RFE	Recursive Feature Elimination
SAR	Structure–Activity Relationship
SBDD	Structure-Based Drug Design
SVM	Support Vector Machines
TKI	Tyrosine Kinase Inhibitor
