# Supplementary material for: Rational Method for Structural Simplification as Key Step in Hit Discovery: The Case of FGFR2 and IGF1R Dual Inhibitors
Source: Int J Mol Sci. 2025 May 7;26(9):4457. doi: 10.3390/ijms26094457 (PMC12072306; doi:10.3390/ijms26094457)
Supplement: Supplementary file 1 [file ijms-26-04457-s001.zip › ijms-3571404-supplementary.pdf]

Rational Method for Structural Simplification as Key Step in Hit Discovery: A case of FGFR2 and IGF1R dual inhibitors.

Endika Torres-Urtizberea [1], José I. Borrell [1], Raimon Puig de la Bellacasa [1] and Roger Estrada-Tejedor [1,*]

[1] Grup de Química Farmacèutica (GQF). IQS School of Engineering, Universitat Ramon Lull, Via Augusta 390, 08017, Barcelona, Spain.

* Correspondence: roger.estrada@iqs.url.edu;

Supporting Information

Combinatorial Library 2

Prediction model details 3

Validation of the models 4

Characterization of synthesized compounds 5

Synthesis of methyl 2-(triphenyl-λ^5-phosphaneylidene)acetate 6

Synthesis of 1-(4-bromophenyl)guanidinium nitrate 6

Synthesis of methyl (*E*)-3-(quinolin-4-yl)acrylate 7

Synthesis of 2-methoxy-6-oxo-4-(quinolin-4-yl)-1,4,5,6-tetrahydropyridine-3-carbonitrile 7

Synthesis of 2-amino-3-(4-bromophenyl)-4-imino-5-(quinolin-4-yl)-4,5,6,8-tetrahydropyrido[2,3-*d*]pyrimidin-7(3*H*)-one 8

Synthesis of 4-amino-2-((4-bromophenyl)amino)-5-(quinolin-4-yl)-5,8-dihydropyrido[2,3-*d*]pyrimidin-7(6*H*)-one 9

Synthesis of 4-amino-2-((4-bromophenyl)amino)-5-(quinolin-4-yl)pyrido[2,3-*d*]pyrimidin-7(8*H*)-one ... 10

Synthesis of 4-amino-2-((4-bromophenyl)amino)-5,8-dihydropyrido[2,3-*d*]pyrimidin-7(6*H*)-one 10

Synthesis of 4-amino-2-((4-bromophenyl)amino)-6-iodopyrido[2,3-*d*]pyrimidin-7(8*H*)-one 11

Synthesis of 4-amino-2-((4-bromophenyl)amino)-6-(3,5-dimethoxyphenyl)pyrido[2,3-*d*]pyrimidin-7(8*H*)-one 12

Synthesis of 2-((4-bromophenyl)amino)-5,8-dihydropyrido[2,3-*d*]pyrimidine-4,7(3*H*,6*H*)-dione 12

Synthesis of 2-((4-bromophenyl)amino)-7-oxo-5,6,7,8-tetrahydropyrido[2,3-*d*]pyrimidin-4-yl trifluoromethanesulfonate 13

Synthesis of 2-((4-bromophenyl)amino)-4-((4-methoxyphenyl)amino)-5,8-dihydropyrido[2,3-*d*]pyrimidin-7(6*H*)-one 14

Biological evaluation 15

References 16

Combinatorial Library

The combinatorial database was performed using the scaffold shown in **Figure S1**.

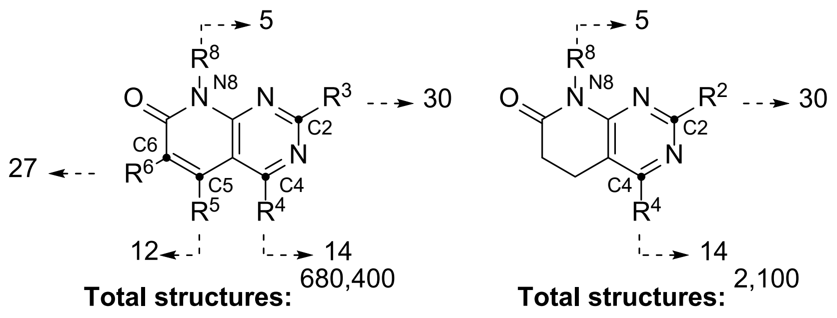

Figure S1 Used scaffold in the first combinatorial library.

For each position, the substitutions were chosen based on synthetic feasibility their introduction based on the general synthetic path, the structural diversity of the groups, and availability of reagents.

For this, *in-house* reagent libraries and different commercial houses (Merk, BLDPharm, FluoroChem, and ABCR) were analyzed, selecting the reagents required for the introduction of the substituents in each position. These reagents consisted of:

- All the **methyl 2-substituted acetates** and **methyl 2-substitutedacrylates** for $\underline{R^6}$ position.
- All **aldehydes** for $\underline{R^5}$ position.
- All **anilines** and **aryl guanidines** for $\underline{R^2}$ position.
- All **alkyl halides** for $\underline{R^8}$ position.
- All **anilines** and **alkyl amines** for $\underline{R^4}$ position.

Among these, those that would present synthetic problems for their introduction were discarded.

- For R^6 and R^5, all substituents bear either acid groups or base/heat sensible groups.
- For R^2 electron deficient anilines.
- For R^8 all groups that do not contain a CH_2 group between the halide and the substitution, multiple halogens, or acidic protons.
- For R^4 electron-deficient nucleophiles.

Finally, a reduced selection of the substituents was done focusing on reagent price and structural diversity, all this considering the final size of the combinatorial database, as the exponential growth of a combinatorially created database could be problematic in feature calculation and selection.

Prediction model details

The prediction models optimized in this work used the feature and hyperparameter sets described afterwards. Both were based on the Supported Vector-Regressor supervised learning method:

FGFR2

- Hyperparameters:
  - gamma: auto
  - C: 1
  - Kernel: rbf
  - tol: 1e-05
  - epsilon: 0.1
- Features:
  - 'a_nF', 'a_nN', 'a_nP', 'a_nS', 'BCUT_SLOGP_3', 'b_max1len', 'b_rotN', 'b_triple', 'chiral', 'density', 'diameter', 'GCUT_PEOE_1', 'GCUT_SLOGP_1', 'GCUT_SMR_3', 'h_pKb', 'h_pstates', 'h_pstrain', 'logP(o/w)', 'PEOE_VSA+0', 'PEOE_VSA+1', 'PEOE_VSA+2', 'PEOE_VSA+5', 'PEOE_VSA-1', 'PEOE_VSA_FPNEG', 'PEOE_VSA_NEG', 'petitjeanSC', 'Q_RPC+', 'Q_VSA_HYD', 'Q_VSA_NEG', 'rings', 'RPC-', 'SlogP_VSA0', 'SlogP_VSA2', 'SlogP_VSA4', 'SlogP_VSA5', 'SlogP_VSA9', 'SMR_VSA1', 'SMR_VSA2', 'BCUT_SLOGP_1', 'a_aro', 'apol', 'a_count', 'b_count', 'chi0v_C', 'mr', 'chi0_C', 'KierA2', 'a_nH', 'GCUT_SMR_0', 'GCUT_SLOGP_3', 'radius', 'a_base', 'chi1_C', 'BCUT_SMR_0', 'Q_VSA_FPOS', 'opr_brigid', 'h_pKa', 'Q_VSA_FPOL', 'chi1v_C', 'BCUT_SMR_1'

IGF1R

- Hyperparameters:
  - gamma: scale, '
  - C: 16
  - kernel: rbf
  - tol: 1e-05
  - epsilon: 0.01
- Features:
  - 'ast_fraglike', 'a_nBr', 'a_nF', 'a_nP', 'a_nS', 'BCUT_SMR_1', 'b_ar', 'b_max1len', 'chiral_u', 'GCUT_PEOE_0', 'GCUT_PEOE_1', 'GCUT_SLOGP_1', 'GCUT_SMR_3', 'h_emd_C', 'h_logD', 'h_pavgQ', 'lip_acc', 'mutagenic', 'opr_leadlike', 'PEOE_VSA+3', 'PEOE_VSA+4', 'PEOE_VSA+5', 'PEOE_VSA+6', 'PEOE_VSA-2', 'Q_VSA_FPOL', 'rsynth', 'SlogP_VSA4', 'SlogP_VSA5', 'SlogP_VSA6', 'SlogP_VSA8', 'SMR_VSA1', 'SMR_VSA3', 'SMR_VSA4', 'SMR_VSA5', 'SMR_VSA7', 'vsa_other', 'vsa_don', 'PEOE_VSA+2', 'PEOE_VSA-1', 'rings', 'PEOE_VSA_FPOL', 'a_acc', 'BCUT_SMR_2', 'a_donacc', 'balabanJ', 'BCUT_PEOE_1', 'Q_VSA_HYD', 'BCUT_SLOGP_3', 'opr_nring', 'h_log_pbo', 'a_nN', 'a_ICM', 'GCUT_SLOGP_0'

Validation of the models

Correlation coefficient values for the validation of SVM model against the target proteins in the validation set: LOO (Q^2), CV5 (R^2 CV5), CV10 (R^2 CV10) and y-randomization (y-rand).

	FGFR2			***IGF1R***		
**Run**	**CV5**	**CV10**	**yrand**	**CV5**	**CV10**	**yrand**
1	0.6836	0.7150	-0.2235	0.6903	0.6951	-0.4545
2	0.6908	0.7239	-0.3902	0.7230	0.7248	-0.3459
3	0.6931	0.7157	-0.1978	0.6866	0.7213	-0.2648
4	0.6681	0.7217	-0.2976	0.7171	0.7177	-0.4095
5	0.6859	0.7156	-0.3153	0.6859	0.7292	-0.3208
6	0.6927	0.7186	-0.2776	0.6459	0.6784	-0.3115
7	0.6947	0.7256	-0.2966	0.7162	0.6818	-0.4369
8	0.6766	0.7196	-0.3308	0.7013	0.7361	-0.2767
9	0.7059	0.7264	-0.2788	0.7068	0.7270	-0.3781
10	0.7091	0.7175	-0.2384	0.7108	0.6862	-0.3252
**Average**	**0.690**	**0.720**	**-0.285**	**0.698**	**0.710**	**-0.352**
**Std. Dev.**	**0.012**	**0.004**	**0.053**	**0.022**	**0.021**	**0.062**

Characterization of synthesized compounds

Instrumentation

Nuclear Magnetic Resonance spectra (^{1}H-NMR, ^{13}C-NMR and ^{19}F-NMR) were recorded on a Varian 400-MR spectrometer (^{1}H-NMR at 400 MHz, ^{13}C-NMR at 100.5 MHz and ^{19}F-NMR at 376 MHz). Chemical shifts are reported in part per million (ppm) on the δ scale; and are referenced to the residual signal from the chosen solvent (DMSO-d_6, 2.50 ppm in ^{1}H-NMR and 39.5 ppm in ^{13}C-NMR. $CDCl_3$, 7.26 ppm in ^{1}H-NMR and 77.2 ppm in ^{13}C-NMR). Coupling constants are reported in Hertz (Hz). Spectral splitting patterns are designated as: s (singlet), d (doublet), dd (double doublet), t (triplet), q (quartet), m (complex multiplet) and * (signal assignment exchangeable).

Infrared Spectra (IR) were recorded in a Thermo Scientific Nicolet iS10 FTIR spectrophotometer with Smart iTr. Values are reported in wave numbers (cm^{-1}) and the notation used is ATR (Attenuated total reflectance). Each described molecular vibrations are designated as: st (stretching), st as (asymmetric stretching), st sy (symmetric stretching) and δ oop (out of the plane deformation).

Elemental microanalyses were obtained on a EuroVector Instruments Euro EA 3000 elemental analyzer.

Microwave irradiation experiments were carried out in an Initiator and initiator+ (Biotage®) microwave apparatus, operating at a frequency of 2.45 GHz with continuous irradiation power from 0 to 400 W. Reactions were carried out in 5 mL, 10 mL or 20 mL glass tubes, sealed with aluminum/Teflon crimp tops, which can be exposed up to 250 °C and 20 bar internal pressure. Temperature was measured with an IR sensor on the outer surface of the process vial and after the irradiation period, the reaction vessel was cooled rapidly to 50 °C by air jet cooling.

Melting points (MP) were determined on a Bibby Scientific Stuart SMP3 Model Melting Point Apparatus and the values are uncorrected.

Mass Spectrometry (MS) was conducted on an Agilent Technologies 5975 quadrupole mass spectrometer operating in electron ionization (EI) mode at 70 eV at IQS. Peaks are reported as m/z followed by their percentage abundance.

High-Resolution Mass Spectrometry (HRMS) data were obtained by using a X500B SCIEX QTOF high-resolution mass spectrometer, using a flow injection analysis (FIA) and electronic spray ionization (ESI).

Automatic flash chromatography was performed in an Isco Combiflash medium-pressure liquid chromatograph with RediSep® silica gel columns (35-70 μm)

Solvents and chemicals, unless otherwise mentioned, were purchased from commercial vendors (Sigma Aldrich, ABCR, Activate Scientific, Alfa Aesar, Apollo Scientific, BLDpharm, Fluorochem and ACROS Organics) and used without further purification.

Synthesis of methyl 2-(triphenyl-λ^5-phosphaneylidene)acetate

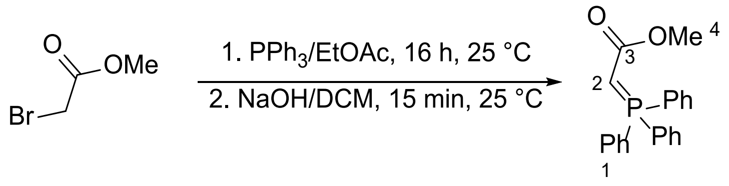

To a solution of 13.755 g (52.5 mmol, 1.05 equiv.) of triphenylphosphine in 80 mL (1.6 mL/mmol bromo derivative) of EtOAc, a solution containing 7.650 g (50 mmol, 1 equiv.) of methyl 2-bromoacetate in 15 mL (0.3 mL/mmol bromo derivative) of EtOAc was added, and the resulting mixture was stirred at RT for 16 h. The resulting suspension was filtered and cleansed with Et_2O. The solid was re-dissolved in 115 mL of DCM, to which 100 mL of concentrated NaOH aqueous solution was added. After reacting the heterogeneous mixture at RT for 15 minutes, the aqueous phase was extracted with DCM, the organic phases were dried using anhydrous $MgSO_4$, filtered, and finally evaporated, affording 14.796 g (46.7 mmol, 89%) of methyl 2-(triphenyl-λ^5-phosphaneylidene)acetate as a white solid.

Spectroscopic Data:

^{1}H-NMR (400 MHz, $CDCl_3$) δ (ppm): 7.70 – 7.40 (m, 15H, H-Ph), 3.51 (s, 3H, H-C4), 2.89 (s, 1H, H-C2).

The recorded spectroscopic data agrees with the structure according to the bibliography[1].

Synthesis of 1-(4-bromophenyl)guanidinium nitrate

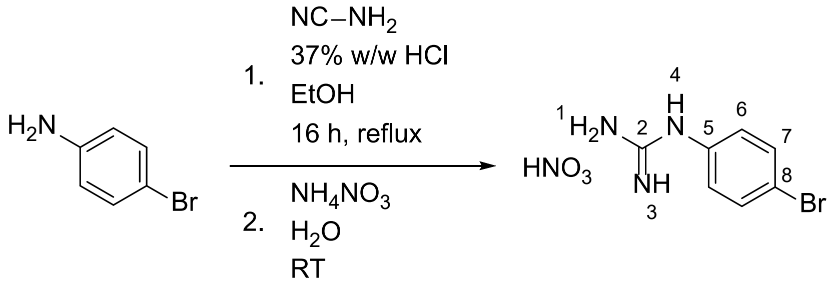

5.1 g (30 mmol, 1 equiv.) of *p*-bromoaniline and 2.646 g (63 mmol, 2.1 equiv.) of cyanamide were dissolved in 30 mL of absolute ethanol. To this solution, 4 mL of 37% w/w HCl were added, and the mixture was refluxed for 16 h. The organics were evaporated completely, and to the remaining sludge, the minimum amount of water was added for all to be dissolved. Subsequently, 5.04 g (63 mmol, 2.1 equiv.) of ammonium nitrate was added in two steps. The formed precipitate was filtered and dried under vacuum over P_2O_5, affording 7.065 g (25.5 mmol, 85%) of product as a white solid.

Spectroscopic Data:

^{1}H-NMR (400 MHz, DMSO-d_6) δ (ppm): 9.64 (s, 1H, H-N4), 7.62 (d, J = 8.8 Hz, 2H, H-C7), 7.44 (s, 4H, H-N3,1), 7.21 (d, J = 8.8 Hz, 2H, H-C6).

^{13}C-NMR (100.5 MHz, DMSO-d_6) δ (ppm): 155.7 (C2), 134.9 (C5), 132.5 (C7), 126.8 (C6), 119.0 (C8).

IR (ATR) ν (cm^{-1}): 3442 (N-H st), 3348, 3314 (N-H st), 3142 (sp^2 C-H st), 1680 (C=N st), 1012 (Bromoaromatics), 817 (sp^2 C-H δ oop), 714 (C-Br st).

HRMS (FIA-ESI-TOF) (m/z): Calculated for $C_7H_9{}^{79}BrN_3$ $[M+H]^+$: 213.9974, found: 213.9968.

MP (°C): 184

Synthesis of methyl (*E*)-3-(quinolin-4-yl)acrylate

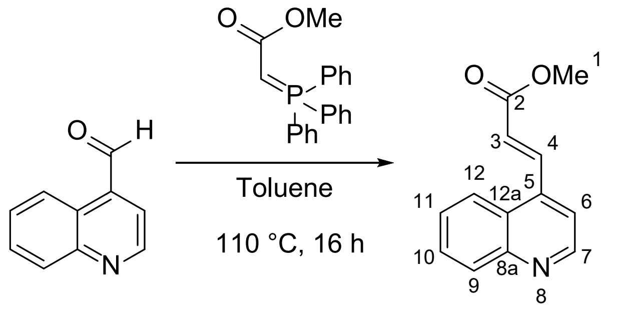

To a solution of 1.570 g (10 mmol, 1 equiv.) of quinoline-4-carbaldehyde in 50 mL toluene, 6.680 g (20 mmol, 2 equiv.) of methyl 2-(triphenyl-λ^5-phosphaneylidene) acetate (2 equiv.) were added. The mixture was refluxed for 16 h. After cooling the mixture, the organic phase was extracted with a 1 M HCl solution. The aqueous phase was then basified with a saturated NaOH solution and extracted with EtOAc. The final organic phase was dried over anhydrous $MgSO_4$, filtered, and evaporated under vacuum, affording 1.656 g (7.77 mmol, 78%) of the product as a white solid.

Spectroscopic Data:

^{1}H-NMR (400 MHz,$CDCl_3$) δ (ppm): 8.94 (d, *J* = 4.4 Hz, 1H, H-C7), 8.42 (d, *J* = 15.6 Hz, 1H, H-C4), 8.18 (ddd, *J* = 11.8, 8.6, 1.3 Hz, 2H, H-C12/9), 7.83 – 7.74 (m, 1H, H-C10*), 7.67 – 7.62 (m, 2H, H-C11*), 7.56 (d, *J* = 4.6 Hz, 1H, H-C6), 6.66 (d, *J* = 16.0 Hz, 1H, H-C3), 3.88 (s, 3H, H-C1).

The recorded spectroscopic data agrees with the structure according to the bibliography[2].

Synthesis of 2-methoxy-6-oxo-4-(quinolin-4-yl)-1,4,5,6-tetrahydropyridine-3-carbonitrile

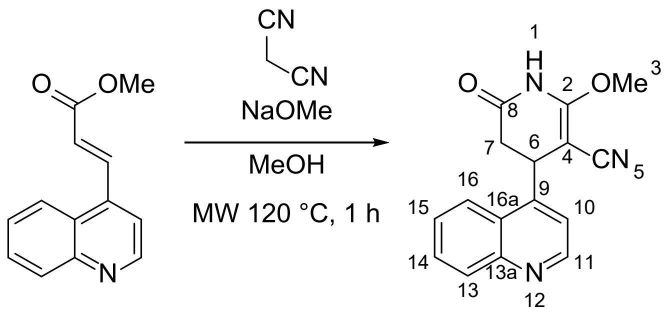

In a microwave vial, 554 mg (8.4 mmol, 1.2 equiv.) of malononitrile and 1.491 mg (7 mmol, 1 equiv.) of methyl (*E*)-3-(quinoline-4-yl)acrylate were dissolved in 14 mL of anhydrous methanol. To this, 605 mg (11.2 mmol, 1.6 equiv.) of sodium methoxide were added and the mixture was heated under microwave irradiation at 120

°C for 1 h. After the vessel was cooled, the remaining dark solution was evaporated, and the remaining sludge was suspended in water and acidified using 2M hydrochloric acid until pH≈5. The precipitated solid was filtered and dried under vacuum over P_2O_5. Finally, the solid was suspended in Et_2O and filtered, yielding 1.602 g (5.74mmol, 82%) of the product as a red solid.

Spectroscopic Data

^{1}H-NMR (400 MHz, DMSO-d_6) δ (ppm): 10.91 (s, 1H, H-N1), 8.90 (d, J = 4.6 Hz, 1H, H-C11), 8.28 (dd, J = 8.5, 1.2 Hz, 1H, H-C16), 8.09 (dd, J = 8.5, 1.3 Hz, 1H, H-C13), 7.82 (ddd, J = 8.2, 6.7, 1.3 Hz, 1H, H-C14), 7.70 (ddd, J = 8.5, 6.8, 1.3 Hz, 1H, H-C15), 7.36 (d, J = 4.5 Hz, 1H, H-C10), 4.90 (dd, J = 7.6, 4.8 Hz, 1H, H-C6), 4.05 (s, 3H, H-C3), 3.16 (dd, J = 16.3, 7.6 Hz, 1H, H-C7), 2.60 (dd, J = 16.2, 4.8 Hz, 1H, H-C7).

^{13}C-NMR (100.5 MHz, DMSO-d_6) δ (ppm): 169.3 (C8), 161.8 (C2), 150.5 (C11), 148.2 (C13a), 145.7 (C9), 130.0 (C13), 129.5 (C14), 127.0 (C15), 125.6 (C16a), 123.5 (C16), 118.6 (C10), 118.1 (C5), 65.8 (C4), 59.0 (C3), 37.4 (C7), 32.3 (C6).

IR (ATR) ν (cm^{-1}): 3061 (sp^2 C-H st), 2874, 2753 (sp^3 C-H st), 2195 (C≡N st), 1701 (C=O st), 1629 (C=C st), 1287 (lactam C-N st), 763 (sp^2 C-H δ oop).

HRMS (FIA-ESI-TOF) (m/z): Calculated for $C_{16}H_{14}N_3O_2$ [M+H]$^+$: 280.1081, found: 280.1075.

MP (°C): 171.

Synthesis of 2-amino-3-(4-bromophenyl)-4-imino-5-(quinolin-4-yl)-4,5,6,8-tetrahydropyrido[2,3-*d*]pyrimidin-7(3*H*)-one

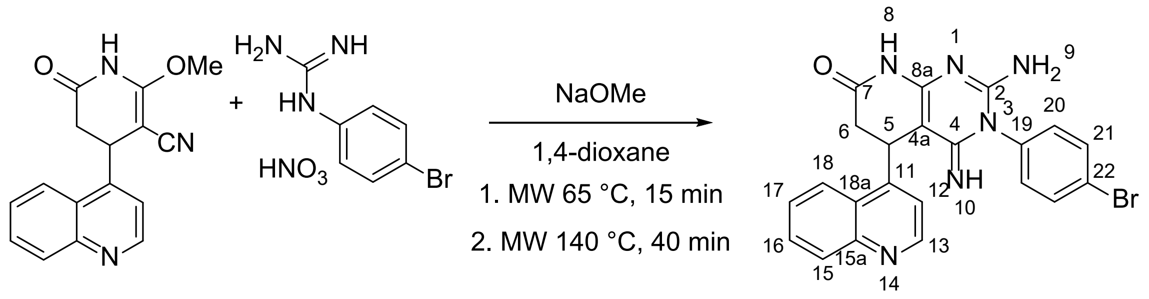

232 mg (4.3 mmol, 4.3 equiv.) of sodium methoxide and 735 mg (3 mmol, 3 equiv.) of 1-(4-bromophenyl)guanidinium nitrate were suspended in 15 mL of anhydrous 1,4-dioxane. The mixture was heated at 65 °C for 15 minutes. After that, the suspended salts were filtered off. To the dioxane solution, 279 mg (1 mmol, 1 equiv.) of 2-methoxy-6-oxo-4-(quinoline-4-yl)-1,4,5,6-tetrahydropyridine-3-carbonitrile were added, and the solution was then heated under microwave irradiation at 140 °C for 40 minutes. The organics were evaporated completely, and to the remaining sludge, the minimum amount of acetone was added and sonicated until a white solid precipitated. Finally, the formed solid was filtered and cleansed with cold acetone yielding 212 mg (0.46 mmol, 46%) of the product as a white solid.

Spectroscopic Data:

IR (ATR) ν (cm^{-1}): 3475, 3313, 3112 (N-H st), 2884 (sp^3 C-H), 1687 (C=O st), 1583, 1525, 1486 (aromatic C-C st), 1069 (Bromoaromatics), 812, 762 (sp^2 C-H δ oop).

HRMS (FIA-ESI-TOF) (m/z): Calculated for $C_{22}H_{18}BrN_6O$ [M+H]$^+$: 461.0720, found: 461.0706.

MP (°C): 220-222.

Due to lack of solubility and inadequate relaxation of spectroscopical signals, ^{1}H-NMR nor ^{13}C-NMR characterization was not possible.

Synthesis of 4-amino-2-((4-bromophenyl)amino)-5-(quinolin-4-yl)-5,8-dihydropyrido[2,3-*d*]pyrimidin-7(6*H*)-one

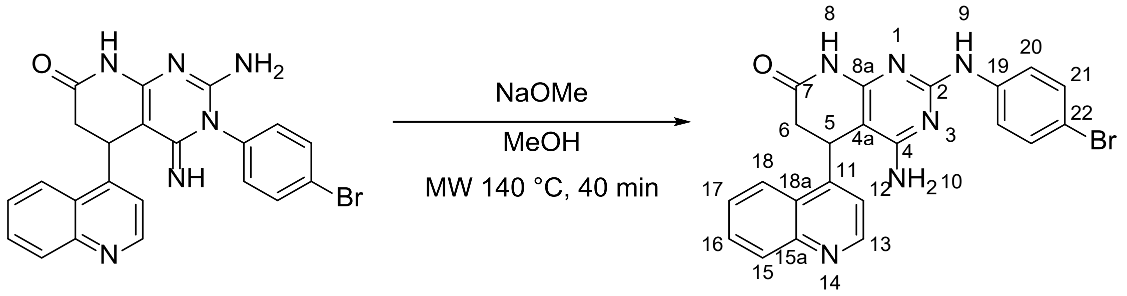

100 mg (1.85 mmol, 1 equiv.) sodium methoxide (1 equiv.) and 854 mg (1.85 mmol, 1 equiv.) of 2-amino-3-(4-bromophenyl)-4-imino-5-(quinoline-4-yl)-4,5,6,8-tetrahydropyrido[2,3-*d*]pyrimidin-7(3*H*)-one were dissolved in 10 mL of anhydrous MeOH in a microwave vial. The mixture was heated at 140 °C under microwave irradiation for 40 minutes. Then, the reaction was cooled and filtered. Finally, the filtrate was rinsed with water and some drops of EtOH and dried under vacuum over P_2O_5, yielding 799 mg (1.73 mmol, 94%) of the product as a white solid

Spectroscopic Data:

^{1}H-NMR (400 MHz, DMSO-*d*$_6$) δ (ppm): 10.37 (s, 1H, H-N8), 9.13 (s, 1H, H-N9), 8.74 (d, *J* = 4.5 Hz, 1H, H-C13), 8.29 (d, *J* = 8.3 Hz, 1H, H-C18), 8.08 (dd, *J* = 8.5, 1.3 Hz, 1H, H-C15), 7.94 – 7.86 (m, 2H, H-C20), 7.81 (ddd, *J* = 8.1, 6.6, 1.3 Hz, 1H, H-C16), 7.72 (ddd, *J* = 8.4, 6.8, 1.4 Hz, 1H, H-C17), 7.41 – 7.31 (m, 2H, H-C21), 6.83 (d, *J* = 4.4 Hz, 1H, H-C12), 6.45 (s, 2H, H-N10), 5.11 (d, *J* = 7.8 Hz, 1H, H-C5), 3.38 – 3.29 (m, 1H, H-C6), 2.61 (d, *J* = 16.0 Hz, 1H, H-C6).

^{13}C-NMR (100.5 MHz, DMSO-*d*$_6$) δ (ppm): 169.6 (C7*), 161.5 (C2*), 158.7 (C4*), 157.8 (C8a*), 150.4 (C13), 148.3 (C15a), 146.8 (C11), 140.8 (C19), 130.8 (C21), 129.9 (C15), 129.2 (C16), 126.4 (C17), 126.0 (C18a), 124.7 (C18), 120.5 (C20), 117.9 (C12), 111.7 (C22), 86.7 (C4a), 38.5 (C6), 30.0 (C5).

IR (ATR) ν (cm^{-1}): 3434, 3293, 3193 (N-H st), 3140, 3079 (sp^2 C-H st), 2976 (sp^3 C-H st), 1677 (C=O st), 1542, 1507, 1438, 1376 (aromatic C-C st), 1243 (lactam C-N st), 813, 794, 768 (sp^2 C-H δ oop).

HRMS (FIA-ESI-TOF) (m/z): Calculated for $C_{22}H_{18}BrN_6O$ $[M+H]^+$: 461.0720, found: 461.0716.

MP (°C): >250.

Synthesis of 4-amino-2-((4-bromophenyl)amino)-5-(quinolin-4-yl)pyrido[2,3-*d*]pyrimidin-7(8*H*)-one

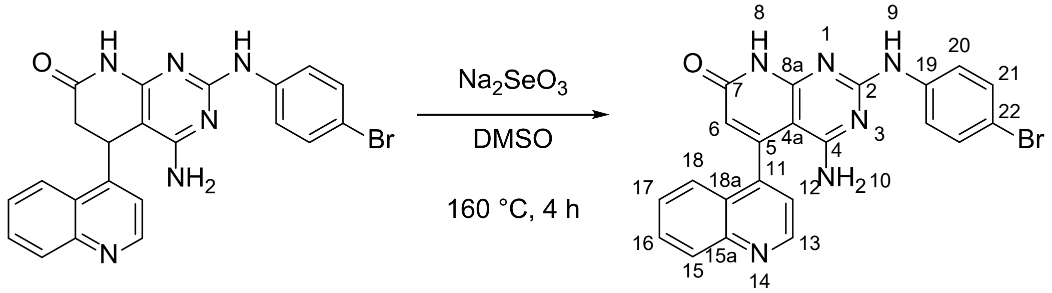

46 mg (0.1 mmol, 1 equiv.) of 4-amino-2-((4-bromophenyl)amino)-5-(quinolin-4-yl)-5,8-dihydropyrido[2,3-*d*]pyrimidin-7(6*H*)-one and 19 mg (0.11 mmol, 1.1 equiv.) of Na_2SeO_3 were dissolved in 0.5 mL of anhydrous DMSO, and the solution was heated at 160 °C for 4 h assisted by microwave irradiation. Then, the mixture was cooled down to room temperature and dropped onto brine. Finally the formed solid was filtered and cleansed with H_2O and EtOH. The solid was dried under vacuum over P_2O_5, yielding 32 mg (0.069 mmol, 69%) of product as a light brown solid.

Spectroscopic Data:

^{1}H-NMR (400 MHz, DMSO-*d*$_6$) δ (ppm): 11.98 (s, 1H), 9.45 (s, 1H), 9.05 (d, *J* = 4.3 Hz, 1H), 8.14 (d, *J* = 8.5 Hz, 1H), 7.94 – 7.79 (m, 3H), 7.71 (d, *J* = 8.3 Hz, 1H), 7.66 – 7.56 (m, 2H), 7.41 – 7.34 (m, 2H), 5.95 (d, *J* = 2.0 Hz, 1H).

IR (ATR) ν (cm^{-1}): 3462, 3361 (N-H st), 3060 (sp^2 C-H st), 1667 (C=O st), 1554, 1440, 1376 (aromatic C-C st), 826, 758, 738 (sp^2 C-H δ oop).

HRMS (FIA-ESI-TOF) (m/z): Calculated for $C_{22}H_{16}{}^{79}BrN_6O^+$ $[M+H]^+$: 459.0563, found: 459.0564.

MP (°C): >250.

Due to lack of solubility ^{13}C-NMR characterization was not possible.

Synthesis of 4-amino-2-((4-bromophenyl)amino)-5,8-dihydropyrido[2,3-*d*]pyrimidin-7(6*H*)-one

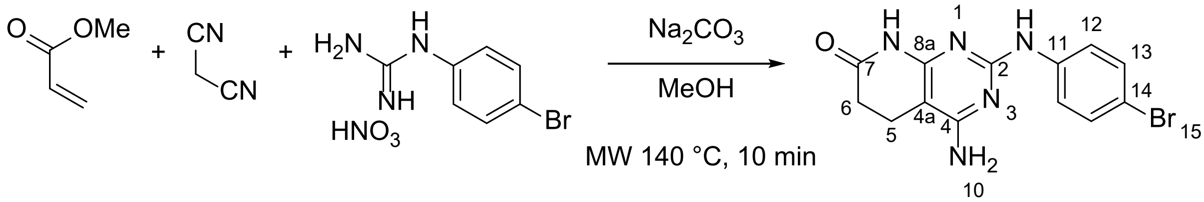

90 mg (1.01 mmol, 1.01 equiv.) of methyl acrylate, 73 mg (1.1 mmol, 1.1 equiv.) malononitrile, 277 mg (1 mmol, 1 equiv.) of 1-(4-bromophenyl)guanidine nitrate, 106 mg (1 mmol, 1 equiv.) of sodium carbonate and 2 mL anhydrous MeOH were mixed and heated at 140°C under microwave irradiation for 10 minutes. After

cooling the reaction down, the formed precipitate was filtered and cleansed with water, EtOH, and Et_2O. Yielding 135 mg (0.4 mmol, 40%) of the product as a white solid.

Spectroscopic Data:

^{1}H-NMR (400 MHz, DMSO-*d₆*) δ (ppm): 10.07 (s, 1H, H-N8), 8.91 (s, 1H, H-N9), 7.83 (d, *J* = 9.0 Hz, 2H, H-C13), 7.31 (d, *J* = 9.0 Hz, 2H, H-C12), 6.41 (s, 2H, H-N10), 2.60 – 2.55 (m, 2H, H-C5), 2.52 – 2.47 (m, *J* = 3.8, 1.9 Hz, 2H, H-C6).

The recorded spectroscopic data agrees with the structure according to the bibliography[3].

Synthesis of 4-amino-2-((4-bromophenyl)amino)-6-iodopyrido[2,3-*d*]pyrimidin-7(8*H*)-one

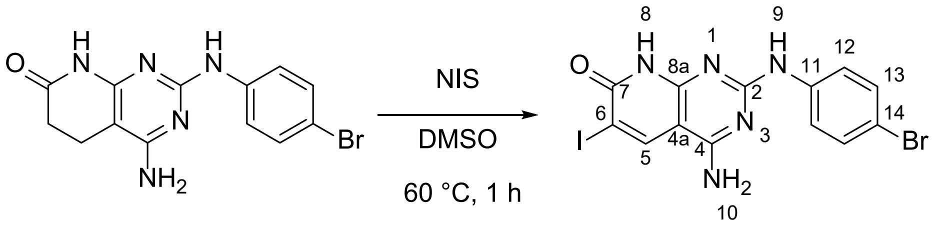

3.600 g (10.8 mmol, 1 equiv.) of 4-amino-2-((4-bromophenyl)amino)-5,8-dihydropyrido[2,3-*d*]pyrimidin-7(6*H*)-one, 4.860 g (21.6 mmol, 2 equiv.) of NIS were dissolved in 40 mL of anhydrous DMSO. The solution was heated at 60 °C for 1 h. The mixture was cooled and dropped into a saturated aqueous solution of sodium thiosulphate. The formed solid was filtered and cleaned with H_2O and EtOH, yielding 4.754 g (10.4 mmol, 96%) of the product as a yellow solid.

Spectroscopic Data:

^{1}H-NMR (400 MHz, DMSO-d_6) δ (ppm): 12.01 (s, 1H, H-N8), 9.43 (s, 1H, H-N9), 8.71 (s, 1H, H-C5), 7.88 (d, *J* = 9.0 Hz, 2H, H-C12), 7.42 (s, 2H, H-N10), 7.37 (d, *J* = 9.0 Hz, 2H, H-C13).

^{13}C-NMR (100.5 MHz, DMSO-d_6) δ (ppm): 160.5 (C7*), 159.9 (C8a*), 159.1 (C2*), 156.1 (C4), 143.3 (C5), 140.0 (C11), 130.9 (C13), 121.3 (C12), 112.8 (C14), 93.7 (C4a**), 84.3 (C6**).

IR (ATR) ν (cm^{-1}): 3640, 3493, 3350 (N-H st), 3079, 2879 (sp^2 C-H st), 1607 (C=O st), 1438 (pyrimidine skeletal), 1073 (bromoaromatics), 1003 (sp_2 C=C-H δ oop), 823 (sp^2 C-H δ oop).

HRMS (FIA-ESI-TOF) (m/z): Calculated for $C_{13}H_{10}BrIN_5O$ $[M+H]^+$: 457.9108, found: 457.9112.

MS (EI, 70 eV) m/z (%): 459.0 (94) $[C_{13}H_9{}^{81}BrIN_5O]^+$, 457.0 (100) $[C_{13}H_9{}^{79}BrIN_5O]^+$, 198.0 (17) $[C_7H_6{}^{81}BrN_2$, *p*-Br aniline derivative$]^+$, 196.0 (19) $[C_7H_6{}^{79}BrN_2$, *p*-Br aniline derivative$]^+$, 189.1 (26), 79.1 (19).

MP (°C): >250.

Synthesis of 4-amino-2-((4-bromophenyl)amino)-6-(3,5-dimethoxyphenyl)pyrido[2,3-*d*]pyrimidin-7(8*H*)-one

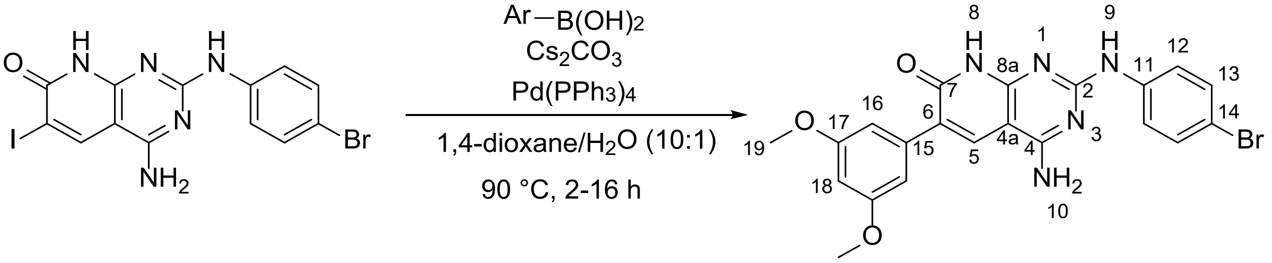

69 mg (0.15 mmol,1 equiv.) of 4-amino-2-((4-bromophenyl)amino)-6-iodopyrido[2,3-*d*]pyrimidin-7(8*H*)-one, 38 mg (0.21 mmol, 1.4 equiv.) of 3,5-dimethoxyphenylboronic acid, 171 mg (0.52 mmol, 3.5 equiv.) of cesium carbonate and 4 mg (0.003 mmol, 0.02 equiv., 2%) of tetrakis(triphenylphosphine)palladium(0) were added in a Schlenk flask. 1.5 mL of degassed 1,4-dioxane/H_2O (10:1) was added. The mixture was heated at 90 °C for 16 h. After cooling the reaction, the suspension was dropped into water and then filtered. The obtained solid was cleansed with H_2O and EtOH, yielding 44 mg (0.09 mmol, 63%) of the product as a pale yellow solid.

Spectroscopic Data:

^{1}H-NMR (400 MHz, DMSO-*d*$_6$) δ (ppm): 11.85 (s, 1H, H-N8), 9.46 (s, 1H, H-N9), 8.30 (s, 1H, H-C5), 7.96 – 7.86 (m, 2H, H-C12), 7.47 (s, 2H, H-C10), 7.42 – 7.34 (m, 2H, H-C13), 6.97 (d, *J* = 2.3 Hz, 2H, H-C16), 6.47 (t, *J* = 2.3 Hz, 1H, H-C18), 3.79 (s, 6H, H-C19).

^{13}C-NMR (100.5 MHz, DMSO-*d*$_6$) δ (ppm): 162.4 (C7*), 161.1 (C4*), 160.0 (C17), 159.0 (C2), 155.1 (C8a*), 140.2 (C11), 138.3 (C15), 133.0 (C5), 130.9 (C13), 123.9 (C6), 121.2 (C12), 112.6 (C14), 106.6 (C16), 98.9 (C18), 92.0 (C4a), 55.2 (C19).

IR (ATR) ν (cm^{-1}): 3597, 3337 (N-H st), 3223, 3057 (sp^2 C-H st), 2992, 2838 (sp^3 C-H st), 1636 (NH_2 δ), 1600 (C=O st), 1435 (pyrimidine skeletal), 1155 (C-O-C st sy), 826, 804 (sp^2 C-H δ oop).

HRMS (FIA-ESI-TOF) (m/z): Calculated for $C_{21}H_{19}{}^{79}BrN_5O_3$ [M+H]$^+$: 468.0666, found: 468.0659.

MP (°C): >250.

Synthesis of 2-((4-bromophenyl)amino)-5,8-dihydropyrido[2,3-*d*]pyrimidine-4,7(3*H*,6*H*)-dione

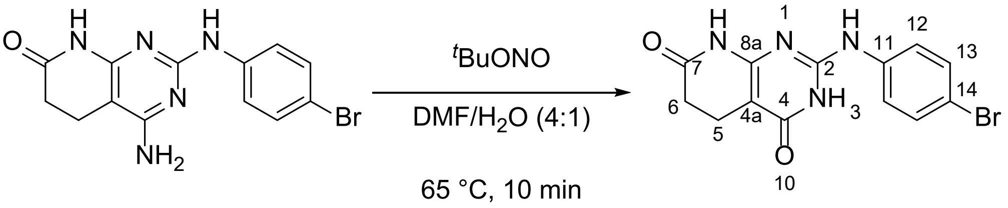

3.015 g (9 mmol, 1 equiv.) of 4-amino-2-((4-bromophenyl)amino)-5,8-dihydropyrido[2,3-*d*]pyrimidin-7(6*H*)-one and 5.88 mL (49.5 mmol) of ^{t}BuONO were dissolved in 42 mL of DMF and 12 mL of H_2O. The mixture was heated at 65 °C for 10 minutes, and after cooling it to room temperature, H_2O was added followed by sonication. The formed precipitate was filtered and cleansed with more H_2O, EtOH and Et_2O doprs, yielding 2.620 g (7.8 mmol, 87%) of the product as a crème colored solid.

Spectroscopic Data:

^{1}H-NMR (400 MHz, DMSO-*d*$_6$) δ (ppm): 10.49 (s, 1H, H-N3), 10.23 (s, 1H, H-N8), 8.89 (s, 1H, H-N9), 7.68 (d, *J* = 9.0 Hz, 2H, H-C12), 7.42 (d, *J* = 8.7 Hz, 2H, H-C13), 2.56 – 2.50 (m, 2H, H-C5), 2.47 – 2.41 (m, 2H, H-C6).

^{13}C-NMR (100.5 MHz, DMSO-*d*$_6$) δ (ppm): 171.5 (C7), 161.6 (C4*), 155.7 (C8a*), 150.9 (C2), 138.3 (C11), 131.3 (C13), 121.3 (C12), 113.9 (C14), 90.8 (C4a), 30.3 (C6), 16.4 (C5).

IR (ATR) ν (cm^{-1}): 3394 (N-H st), 3115 (sp^2 C-H st), 1668 (C=O st), 1611, 1505 (pyrimidine skeletal), 1455 (N-H δ ip), 1312 (lactam C-N st), 1001 (bromoaromatic signal), 807 (N-H δ oop), 756 (sp^2 C-H δ oop).

HRMS (FIA-ESI-TOF) (m/z): Calculated for $C_{13}H_{12}{}^{79}BrN_4O_2^+$ [M+H]$^+$: 335.0138, found: 335.0144.

MS (EI, 70 eV) m/z (%): 336.05 (89) [$C_{13}H_{11}{}^{81}BrN_4O_2$]$^+$, 335.05 (73), 334.05 (100) [$C_{13}H_{11}{}^{79}BrN_4O_2$]$^+$, 173 (8) [$C_6H_6{}^{81}BrN$, *p*-Br aniline]$^+$, 171 (10) [$C_6H_6{}^{79}BrN$, *p*-Br aniline]$^+$.

MP (°C): >250.

Synthesis of 2-((4-bromophenyl)amino)-7-oxo-5,6,7,8-tetrahydropyrido[2,3-*d*]pyrimidin-4-yl trifluoromethanesulfonate

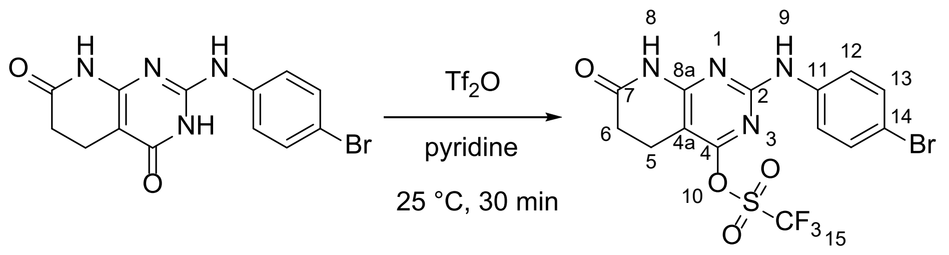

To a solution of 3.350 g (10 mmol, 1 euqiv.) of 2-((4-bromophenyl)amino)-5,8-dihydropyrido[2,3-*d*]pyrimidine-4,7(3*H*,6*H*)-dione in 60 mL of pyridine, 3.36 mL (20 mmol, 2 equiv.) of Tf_2O were added dropwise, and the mixture was stirred at room temperature for 30 minutes. Then, the solution was concentrated to dryness under vacuum to later be diluted with H_2O. The formed solid was filtered and wasched with H_2O and EtOH. The solid is finally digested with more EtOH, re-filtered and cleansed with more EtOH and Et_2O, yielding 3.912 g (8.4 mmol, 84%) of the product as a grey solid.

Spectroscopic Data:

^{1}H-NMR (400 MHz, DMSO-*d*$_6$) δ (ppm): 11.21 (s, 1H, H-N8), 9.97 (s, 1H, H-N9), 7.72 (d, *J* = 9.1 Hz, 2H, H-C13), 7.43 (d, *J* = 8.9 Hz, 2H, H-C12), 2.78 (t, *J* = 7.6 Hz, 2H, H-C5), 2.62 (t, *J* = 7.6 Hz, 2H, H-C6).

^{13}C-NMR (100.5 MHz, DMSO-*d*$_6$) δ (ppm): 171.7 (C7), 162.5 (C8a*), 159.2 (C4*), 157.4 (C2), 139.3 (C11), 131.6 (C13), 121.8 (C12), 118.3 (q, *J* = 320.4 Hz, C15), 114.3 (C14), 94.9 (C4a), 30.0 (C6), 17.1 (C5).

^{19}F-NMR (376 MHz, DMSO-*d*$_6$) δ (ppm): -73.69 (s, F-C15).

IR (ATR) ν (cm^{-1}): 3293 (N-H st), 3125 (sp^2 C-H st), 2987 (sp^3 C-H st), 1692 (C=O), 1625, 1547 (pyrimidine skeletal), 1426 (O=S=O st as), 1220 (O=S=O st sy), 1131 (C-F st), 803, 744 (sp^2 C-H δ oop).

HRMS (FIA-ESI-TOF) (m/z): Calculated for $C_{14}H_{11}{}^{79}BrF_3N_4O_4S^+$ $[M+H]^+$: 466.9631, found: 466.9644.

MS (EI, 70 eV) m/z (%): 468.05 (70) $[C_{14}H_{10}{}^{81}BrF_3N_4O_4S, M]^+$, 467.05 (16), 466.05 (73) $[C_{14}H_{10}{}^{79}BrF_3N_4O_4S, M]^+$, 335.1 (9) $[C_{13}H_{10}{}^{81}BrN_4O_2, -SO_2CF_3]^+$, 254.15 (100) $[C_{13}H_9N_4O_2, -SO_2CF_3 \text{ and } -HBr]^+$.

MP (°C): >250.

Synthesis of 2-((4-bromophenyl)amino)-4-((4-methoxyphenyl)amino)-5,8-dihydropyrido[2,3-*d*]pyrimidin-7(6*H*)-one

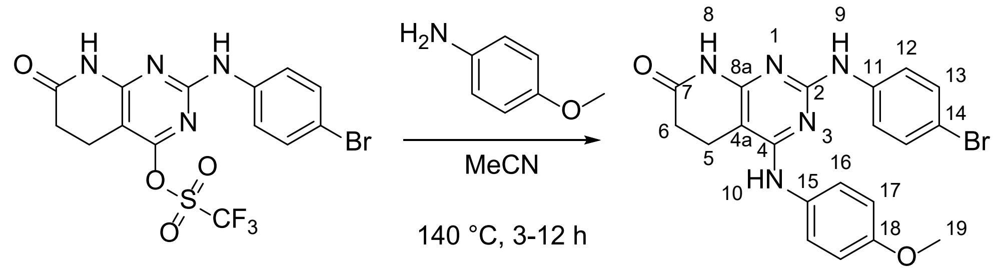

46 mg (0.1 mmol, 1 equiv.) of 2-((4-bromophenyl)amino)-7-oxo-5,6,7,8-tetrahydropyrido[2,3-*d*]pyrimidin-4-yl trifluoromethanesulfonate and 55 mg (0.3 mmol, 3 equiv.) of *p*-anisidine were suspended in 2 mL of anhydrous acetonitrile. The mixture was heated at 140 °C assisted by microwave irradiation for 10 h. Then, the reaction was cooled to room temperature, H_2O was added, and the formed solid was filtered and cleased with H_2O and EtOH. The solid was dried under vacuum over P_2O_5, yielding 40 mg (0.06 mmol, 61%) of product as a white solid.

Spectroscopic Data:

^{1}H-NMR (400 MHz, DMSO-*d₆*) δ (ppm): 10.17 (s, 1H, H-N8), 8.99 (s, 1H, H-N9), 8.29 (s, 1H, H-N10), 7.70 (d, *J* = 8.7 Hz, 2H, H-C12), 7.45 (d, *J* = 9.1 Hz, 2H, H-C16), 7.24 (d, *J* = 8.7 Hz, 2H, H-C13), 6.92 (d, *J* = 9.0 Hz, 2H, H-C17), 3.77 (s, 3H, H-C19), 2.76 (t, *J* = 7.7 Hz, 2H, H-C5), 2.56 (t, *J* = 7.7 Hz, 2H, H-C6).

^{13}C-NMR (100.5 MHz, DMSO-d_6) δ (ppm): 171.3 (C7), 158.5 (C2*), 157.3 (C4*), 156.6 (C8a*), 155.4 (C18), 140.6 (C11), 132.9 (C15), 130.7 (C13), 124.6 (C16), 120.4 (C12), 113.5 (C17), 111.6 (C14), 87.6 (C4a), 55.2 (C19), 30.3 (C6), 17.3 (C5).

IR (ATR) ν (cm^{-1}): 3341, 3288, 3202 (N-H st), 3116 (sp^2 C-H st), 2964, 2834 (sp^3 C-H st), 1671 (C=O st), 1242 (C-O-C st as), 1073 (C-O-C st sy), 824, 799 (sp^2 C-H δ oop).

HRMS (FIA-ESI-TOF) (m/z): Calculated for $C_{20}H_{19}{}^{79}BrN_5O_2^+$ $[M+H]^+$: 440.0717, found: 440.0712.

MP (°C): >250.

Biological evaluation

The kinase inhibition profile of compounds was evaluated at Reaction Biology (https://www.reactionbiology.com/) (accessed on 10 February 2025) by measuring singlicate residual activity values at a concentration of 1 µM of the test compound in front of the several tyrosine kinases (EGFR, FGFR2, IGF1R, PDGFRa, and VEGFR2).

These were performed following the next protocol:

The compounds were dissolved to 1×10^{-3} M stock solutions in 100% DMSO. Subsequently, 100 µL of each stock solution were transferred into wells A3-F12 of a microtiter plate ("master plate"). Wells A1-F2 were filled with 100 µL of 100% DMSO as controls. In total, 5 × 10 µL of the master plate were aliquoted into 5 copy plates, which were stored at −20 °C until use. For the testing of each group of up to 8 kinases, one copy plate was used. In the process, 90 µL of H2O were added to each well of a copy plate. To minimize precipitation, the H_2O was added to each well only a few minutes before the transfer of the compound solutions into the assay plates. The plate was shaken thoroughly, resulting in a "compound dilution plate" with a compound concentration of 1×10^{-4} M/10 % DMSO. This plate was used for the transfer of 5 µL of compound solution into the assay plates. The final volume of the assay was 50 µL. All compounds were tested at 1×10^{-5} M in singlicate. The final DMSO concentration in the reaction cocktails was 1% in all cases. The compound dilution plates were disposed of at the end of each working day.

A radiometric protein kinase assay (^{33}PanQinase® Activity Assay) was used for measuring the kinase activity of the corresponding protein kinases. All kinase assays were performed in 96-well FlashPlatesTM from Perkin Elmer (Boston, MA, USA) in a 50 µL reaction volume. The reaction cocktail was pipetted in 4 steps in the following order: 10µL of non-radioactive ATP solution (in H_2O); 25 µL of assay buffer/ [γ-^{33}P]-ATP mixture; 5 µL of the test sample in 10% DMSO; and 10 µL of enzyme/substrate mixture. The assay for all protein kinases contained 70 mM HEPES-NaOH pH 7.5, 3 mM $MgCl_2$, 3 mM $MnCl_2$, 3 µM Na-orthovanadate, 1.2 mM DTT, ATP (variable amounts, corresponding to the apparent ATP-Km of the respective kinase), [γ-^{33}P]-ATP (approximately 8 × 105 cpm per well), protein kinase (variable amounts), and substrate (variable amounts). The protein kinase reaction cocktails were incubated at 30 °C for 60 min. The reaction was stopped with 50 µL of 2 % (v/v) H_3PO_4, plates were aspirated and washed two times with 200µL of 0.9 % (w/v) NaCl. All assays were performed with a BeckmanCoulter Biomek 2000/SL robotic system. The incorporation of ^{33}Pi (counting of "cpm") was determined with a microplate scintillation counter (Microbeta, Wallac). All protein kinase assays were performed with a BeckmanCoulter Core robotic system.

For each kinase, the median value of the cpm of six wells of column 1 of each assay plate was defined as the "low control" (n = 6). This value reflects the unspecific binding of radioactivity to the plate in the absence of a protein kinase but in the presence of the substrate. Additionally, for each kinase, the median value of the cpm of six wells of column 2 of each assay plate was taken as the "high control", i.e. full activity in the absence of any inhibitor (n = 6). The difference between the high and low control of each enzyme was taken as 100% activity. As part of the data evaluation, the low control of each kinase was subtracted from the high control value as well as from their corresponding "compound values". The residual activity (in %) for each compound well was calculated by using the following formula:

Res. Activity (%) = 100 × [(signal of compound − low control)/(high control − low control)]

As a parameter for assay quality, the Z´-factor30 for the low and high controls of each assay plate (n = 8) was used. Reaction Biology´s criterion for repetition of an assay plate is a Z´-factor below 0.4. Z´-factors did not drop below 0.51, indicating an excellent assay quality.

References

1. Boers, R.B.; Randulfe, Y.P.; Van Der Haas, H.N.S.; Van Rossum-Baan, M.; Lugtenburg, J. Synthesis and Spectroscopic Characterization of 1-13C- and 4-13C-Plastoquinone-9. *European J Org Chem* **2002**, 2094–2108, doi:10.1002/1099-0690(200207)2002:13<2094::AID-EJOC2094>3.0.CO;2-E.

2. El-Batta, A.; Jiang, C.; Zhao, W.; Anness, R.; Cooksy, A.L.; Bergdahl, M. Wittig Reactions in Water Media Employing Stabilized Ylides with Aldehydes. Synthesis of α,β-Unsaturated Esters from Mixing Aldehydes, α-Bromoesters, and Ph3P in Aqueous NaHCO3. *Journal of Organic Chemistry* **2007**, *72*, 5244–5259, doi:10.1021/jo070665k.

3. Galve, I.; Ondoño, R.; De Rocafiguera, C.; Puig De La Bellacasa, R.; Batllori, X.; Puigjaner, C.; Font-Bardia, M.; Vallcorba, O.; Teixidó, J.; Borrell, J.I. A Captured Room Temperature Stable Wheland Intermediate as a Key Structure for the Orthogonal Decoration of 4-Amino-Pyrido[2,3-*d*] Pyrimidin-7(8 H)-Ones. *Org Biomol Chem* **2020**, *18*, 9810–9815, doi:10.1039/d0ob01785j.
